# Ethyl 2-{[5-(3-chloro­phen­yl)-1-phenyl-1*H*-pyrazol-3-yl]­oxy}acetate

**DOI:** 10.1107/S1600536811052937

**Published:** 2011-12-14

**Authors:** Xiao-Li Yang

**Affiliations:** a Department of Applied Chemistry, College of Science, Nanjing University of Technology, Nanjing 210009, People’s Republic of China

## Abstract

The title compound, C_19_H_17_ClN_2_O_3_, was synthesized by the reaction of 5-(3-chloro­phen­yl)-1-phenyl-1*H*-pyrazol-3-ol and ethyl 2-bromo­acetate. In the crystal, the C- and N-linked benzene rings are twisted by 45.15 (3) and 53.55 (3)°, respectively, from the plane of the bridging 1*H*-pyrazole ring.

## Related literature

For 1*H*-pyrazol-3-oxy derivatives, see: Li *et al.* (2010 ▶). For alkyl­oxyacetates as bioactive groups, see: Tohyama & Sanemitsu (2001 ▶). For bond-length data, see: Allen *et al.* (1987 ▶). For the synthetic procedure, see: Liu *et al.* (2011 ▶);
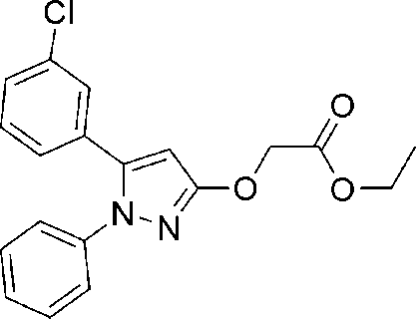


## Experimental

### 

#### Crystal data

C_19_H_17_ClN_2_O_3_
*M* *_r_* = 356.80
Orthorhombic, 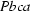

*a* = 11.6158 (11) Å
*b* = 15.9119 (16) Å
*c* = 19.302 (2) Å
*V* = 3567.6 (6) Å^3^
*Z* = 8
Mo *K*α radiation
μ = 0.23 mm^−1^
*T* = 293 K
0.30 × 0.30 × 0.10 mm

#### Data collection

Enraf–Nonius CAD-4 diffractometer
Absorption correction: ψ scan (North *et al.*, 1968 ▶) *T*_min_ = 0.933, *T*_max_ = 0.977
6360 measured reflections
3244 independent reflections
1787 reflections with *I* > 2σ(*I*)
*R*_int_ = 0.092
3 standard reflections every 200 reflections  intensity decay: 1%

#### Refinement

*R*[*F* ^2^ > 2σ(*F* ^2^)] = 0.055
*wR*(*F* ^2^) = 0.093
*S* = 1.01
3244 reflections
226 parameters
H-atom parameters constrained
Δρ_max_ = 0.17 e Å^−3^
Δρ_min_ = −0.17 e Å^−3^

### 

Data collection: *CAD-4 EXPRESS* (Enraf–Nonius, 1994 ▶); cell refinement: *CAD-4 EXPRESS*; data reduction: *XCAD4* (Harms & Wocadlo, 1995 ▶); program(s) used to solve structure: *SHELXS97* (Sheldrick, 2008 ▶); program(s) used to refine structure: *SHELXL97* (Sheldrick, 2008 ▶); molecular graphics: *SHELXTL* (Sheldrick, 2008 ▶); software used to prepare material for publication: *SHELXTL*.

## Supplementary Material

Crystal structure: contains datablock(s) I, global. DOI: 10.1107/S1600536811052937/ez2274sup1.cif

Structure factors: contains datablock(s) I. DOI: 10.1107/S1600536811052937/ez2274Isup2.hkl

Supplementary material file. DOI: 10.1107/S1600536811052937/ez2274Isup3.cml

Additional supplementary materials:  crystallographic information; 3D view; checkCIF report

## Figures and Tables

**Fig. 1.**
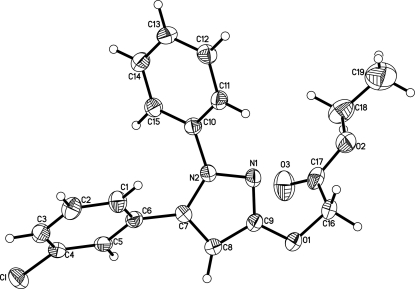
The molecular structure of (I), with the atom-numbering scheme. Displacement ellipsoids are drawn at the 50% probability level.

## supplementary crystallographic information

### Comment

Since the discovery of the strobilurin fungicide pyraclostrobin by BASF scientists, 1*H*-pyrazol-3-oxy derivatives have attracted considerable attention in chemical and medicinal research because of their low mammalian toxicity and diverse bioactivities (Li *et al.*, 2010). Furthermore, several biological studies have also pointed out the value of alkyloxyacetates (Tohyama & Sanemitsu, 2001) as bioactive groups. Recently, focusing on incorporating an alkyloxyacetate group into 1*H*-pyrazol-3-oxy derivatives in the hope of obtaining compounds with potential bioactivities, we report here the crystal structure of the title compound (I).

In the molecule of I (Fig.1) the bond lengths (Allen *et al.*, 1987) and angles are within normal ranges. The C-linked benzene ring A (C1—C6) and N-linked benzene ring B (C10—C15) are twisted 45.15 (3)° and 53.55 (3)° from the plane of the bridge 1*H*-pyrazol ring (N2/N3/C7—C8), respectively. Rings A and B are, of course, planar and the dihedral angle between them is 61.11 (3)°.

### Experimental

The title compound was prepared by the literature method (Liu *et al.*, 2011). Crystals suitable for X-ray analysis were obtained by dissolving I (0.5 g) in ethyl acetate (25 ml) and evaporating the solvent slowly at room temperature for about 10 d.

### Refinement

H atoms were positioned geometrically, with C—H = 0.93, 0.97 and 0.96 Å for aromatic, methylene and methyl H, respectively, and constrained to ride on their parent atoms, with *U*_iso_(H) = *xU*_eq_(C), where *x* = 1.2 for aromatic H, and *x* = 1.5 for other H.

### Crystal data

**Table:**

C_19_H_17_ClN_2_O_3_	*D*_x_ = 1.329 Mg m^−^^3^
*M**_r_* = 356.80	Melting point: 369 K
Orthorhombic, *P**b**c**a*	Mo *K*α radiation, λ = 0.71073 Å
Hall symbol: -P 2ac 2ab	Cell parameters from 25 reflections
*a* = 11.6158 (11) Å	θ = 10–13°
*b* = 15.9119 (16) Å	µ = 0.23 mm^−^^1^
*c* = 19.302 (2) Å	*T* = 293 K
*V* = 3567.6 (6) Å^3^	Block, colourless
*Z* = 8	0.30 × 0.30 × 0.10 mm
*F*(000) = 1488	

### Data collection

**Table:**

Enraf–Nonius CAD-4 diffractometer	1787 reflections with *I* > 2σ(*I*)
Radiation source: fine-focus sealed tube	*R*_int_ = 0.092
graphite	θ_max_ = 25.4°, θ_min_ = 2.1°
ω/2θ scans	*h* = 0→14
Absorption correction: ψ scan (North *et al.*, 1968)	*k* = 0→19
*T*_min_ = 0.933, *T*_max_ = 0.977	*l* = 0→23
6360 measured reflections	3 standard reflections every 200 reflections
3244 independent reflections	intensity decay: 1%

### Refinement

**Table:**

Refinement on *F*^2^	Primary atom site location: structure-invariant direct methods
Least-squares matrix: full	Secondary atom site location: difference Fourier map
*R*[*F*^2^ > 2σ(*F*^2^)] = 0.055	Hydrogen site location: inferred from neighbouring sites
*wR*(*F*^2^) = 0.093	H-atom parameters constrained
*S* = 1.01	*w* = 1/[σ^2^(*F*_o_^2^) + (0.010*P*)^2^] where *P* = (*F*_o_^2^ + 2*F*_c_^2^)/3
3244 reflections	(Δ/σ)_max_ < 0.001
226 parameters	Δρ_max_ = 0.17 e Å^−^^3^
0 restraints	Δρ_min_ = −0.17 e Å^−^^3^

### Special details

**Table:**

Geometry. All e.s.d.'s (except the e.s.d. in the dihedral angle between two l.s. planes) are estimated using the full covariance matrix. The cell e.s.d.'s are taken into account individually in the estimation of e.s.d.'s in distances, angles and torsion angles; correlations between e.s.d.'s in cell parameters are only used when they are defined by crystal symmetry. An approximate (isotropic) treatment of cell e.s.d.'s is used for estimating e.s.d.'s involving l.s. planes.
Refinement. Refinement of *F*^2^ against ALL reflections. The weighted *R*-factor *wR* and goodness of fit *S* are based on *F*^2^, conventional *R*-factors *R* are based on *F*, with *F* set to zero for negative *F*^2^. The threshold expression of *F*^2^ > σ(*F*^2^) is used only for calculating *R*-factors(gt) *etc*. and is not relevant to the choice of reflections for refinement. *R*-factors based on *F*^2^ are statistically about twice as large as those based on *F*, and *R*- factors based on ALL data will be even larger.

### Fractional atomic coordinates and isotropic or equivalent isotropic displacement parameters (Å^2^)

**Table:**

	*x*	*y*	*z*	*U*_iso_*/*U*_eq_	
Cl	0.35737 (6)	0.17569 (5)	0.39581 (4)	0.0686 (3)	
O1	0.00916 (16)	0.53604 (11)	0.21036 (10)	0.0628 (6)	
N1	−0.10819 (17)	0.42944 (13)	0.25540 (13)	0.0491 (6)	
C1	0.0080 (2)	0.28411 (17)	0.43656 (13)	0.0535 (8)	
H1B	−0.0653	0.3054	0.4447	0.064*	
N2	−0.08809 (16)	0.36663 (13)	0.30241 (11)	0.0453 (6)	
O2	−0.18554 (18)	0.49472 (12)	0.07176 (11)	0.0641 (6)	
C2	0.0559 (2)	0.22869 (18)	0.48294 (14)	0.0575 (8)	
H2A	0.0148	0.2126	0.5221	0.069*	
C3	0.1657 (2)	0.19648 (16)	0.47169 (14)	0.0516 (8)	
H3A	0.1989	0.1599	0.5035	0.062*	
O3	−0.0121 (2)	0.43762 (13)	0.09175 (12)	0.0896 (8)	
C4	0.2242 (2)	0.21959 (16)	0.41286 (14)	0.0450 (7)	
C5	0.1774 (2)	0.27566 (16)	0.36660 (13)	0.0421 (7)	
H5A	0.2190	0.2917	0.3276	0.050*	
C6	0.0677 (2)	0.30848 (16)	0.37803 (13)	0.0417 (7)	
C7	0.0204 (2)	0.36878 (17)	0.32760 (13)	0.0423 (6)	
C8	0.0733 (2)	0.43603 (16)	0.29721 (14)	0.0506 (7)	
H8A	0.1480	0.4552	0.3045	0.061*	
C9	−0.0090 (2)	0.46931 (16)	0.25317 (15)	0.0485 (7)	
C10	−0.1769 (2)	0.30433 (16)	0.31081 (14)	0.0417 (7)	
C11	−0.2865 (2)	0.32957 (17)	0.32679 (13)	0.0478 (7)	
H11A	−0.3019	0.3860	0.3351	0.057*	
C12	−0.3740 (2)	0.27127 (18)	0.33060 (15)	0.0530 (8)	
H12A	−0.4489	0.2883	0.3400	0.064*	
C13	−0.3490 (2)	0.18712 (18)	0.32025 (15)	0.0571 (8)	
H13A	−0.4074	0.1473	0.3235	0.068*	
C14	−0.2385 (2)	0.16216 (18)	0.30514 (15)	0.0590 (8)	
H14A	−0.2223	0.1055	0.2987	0.071*	
C15	−0.1520 (2)	0.22070 (16)	0.29954 (14)	0.0492 (7)	
H15A	−0.0775	0.2041	0.2883	0.059*	
C16	−0.0794 (2)	0.55185 (16)	0.16175 (16)	0.0583 (8)	
H16A	−0.0667	0.6065	0.1408	0.070*	
H16B	−0.1526	0.5538	0.1859	0.070*	
C17	−0.0859 (3)	0.48651 (18)	0.10533 (17)	0.0572 (8)	
C18	−0.2018 (3)	0.4426 (2)	0.01132 (19)	0.0866 (11)	
H18A	−0.2028	0.3837	0.0243	0.104*	
H18B	−0.1396	0.4514	−0.0215	0.104*	
C19	−0.3146 (4)	0.4671 (3)	−0.0204 (2)	0.1339 (17)	
H19A	−0.3283	0.4337	−0.0609	0.201*	
H19B	−0.3124	0.5255	−0.0330	0.201*	
H19C	−0.3753	0.4580	0.0125	0.201*	

### Atomic displacement parameters (Å^2^)

**Table:**

	*U*^11^	*U*^22^	*U*^33^	*U*^12^	*U*^13^	*U*^23^
Cl	0.0436 (4)	0.0759 (5)	0.0863 (6)	0.0071 (4)	−0.0079 (4)	0.0128 (5)
O1	0.0566 (12)	0.0455 (11)	0.0863 (15)	−0.0094 (10)	−0.0137 (12)	0.0259 (12)
N1	0.0422 (13)	0.0448 (13)	0.0601 (15)	0.0010 (11)	−0.0036 (11)	0.0165 (14)
C1	0.0543 (18)	0.0535 (18)	0.0527 (17)	0.0112 (15)	0.0052 (16)	0.0014 (17)
N2	0.0385 (12)	0.0426 (13)	0.0549 (15)	−0.0003 (11)	−0.0041 (11)	0.0109 (13)
O2	0.0703 (14)	0.0519 (12)	0.0701 (14)	0.0005 (11)	−0.0086 (13)	−0.0037 (12)
C2	0.071 (2)	0.0544 (19)	0.0469 (17)	0.0013 (17)	0.0090 (16)	0.0051 (17)
C3	0.065 (2)	0.0460 (18)	0.0441 (18)	−0.0032 (15)	−0.0114 (16)	0.0000 (15)
O3	0.0931 (17)	0.0774 (16)	0.0982 (18)	0.0382 (14)	0.0138 (16)	−0.0003 (16)
C4	0.0440 (16)	0.0420 (16)	0.0491 (17)	−0.0059 (13)	−0.0125 (14)	−0.0014 (15)
C5	0.0380 (15)	0.0440 (16)	0.0442 (15)	−0.0095 (12)	−0.0039 (13)	−0.0002 (15)
C6	0.0453 (16)	0.0393 (16)	0.0404 (16)	−0.0041 (13)	−0.0033 (13)	0.0008 (13)
C7	0.0386 (14)	0.0437 (15)	0.0446 (16)	0.0019 (14)	−0.0038 (13)	0.0015 (15)
C8	0.0428 (16)	0.0414 (16)	0.0676 (19)	−0.0053 (14)	−0.0091 (15)	0.0080 (16)
C9	0.0451 (16)	0.0377 (15)	0.0628 (19)	−0.0017 (13)	−0.0007 (16)	0.0104 (16)
C10	0.0382 (15)	0.0425 (16)	0.0444 (16)	−0.0019 (13)	0.0037 (13)	0.0046 (14)
C11	0.0470 (16)	0.0405 (15)	0.0560 (17)	0.0049 (14)	0.0008 (15)	0.0019 (16)
C12	0.0389 (16)	0.063 (2)	0.0577 (19)	0.0015 (15)	0.0088 (15)	0.0056 (18)
C13	0.0544 (18)	0.0512 (19)	0.066 (2)	−0.0127 (16)	−0.0039 (18)	0.0034 (17)
C14	0.0554 (18)	0.0421 (16)	0.079 (2)	−0.0018 (15)	−0.0007 (18)	0.0016 (19)
C15	0.0417 (15)	0.0468 (17)	0.0591 (18)	0.0063 (14)	0.0032 (15)	−0.0018 (16)
C16	0.0614 (19)	0.0359 (16)	0.078 (2)	0.0010 (15)	−0.0115 (18)	0.0153 (18)
C17	0.068 (2)	0.0416 (18)	0.062 (2)	0.0019 (17)	0.0100 (19)	0.0162 (19)
C18	0.111 (3)	0.067 (2)	0.082 (3)	−0.014 (2)	0.002 (2)	−0.013 (2)
C19	0.161 (4)	0.118 (4)	0.122 (4)	−0.023 (3)	−0.041 (4)	−0.019 (3)

### Geometric parameters (Å, °)

**Table:**

Cl—C4	1.729 (3)	C8—C9	1.385 (3)
O1—C9	1.362 (3)	C8—H8A	0.9300
O1—C16	1.415 (3)	C10—C11	1.370 (3)
N1—C9	1.316 (3)	C10—C15	1.379 (3)
N1—N2	1.370 (3)	C11—C12	1.378 (3)
C1—C2	1.374 (3)	C11—H11A	0.9300
C1—C6	1.381 (3)	C12—C13	1.384 (4)
C1—H1B	0.9300	C12—H12A	0.9300
N2—C7	1.351 (3)	C13—C14	1.375 (3)
N2—C10	1.440 (3)	C13—H13A	0.9300
O2—C17	1.333 (3)	C14—C15	1.375 (3)
O2—C18	1.444 (3)	C14—H14A	0.9300
C2—C3	1.391 (4)	C15—H15A	0.9300
C2—H2A	0.9300	C16—C17	1.508 (4)
C3—C4	1.373 (3)	C16—H16A	0.9700
C3—H3A	0.9300	C16—H16B	0.9700
O3—C17	1.188 (3)	C18—C19	1.498 (4)
C4—C5	1.374 (3)	C18—H18A	0.9700
C5—C6	1.394 (3)	C18—H18B	0.9700
C5—H5A	0.9300	C19—H19A	0.9600
C6—C7	1.473 (3)	C19—H19B	0.9600
C7—C8	1.367 (3)	C19—H19C	0.9600
			
C9—O1—C16	115.3 (2)	C10—C11—C12	120.0 (3)
C9—N1—N2	103.0 (2)	C10—C11—H11A	120.0
C2—C1—C6	120.6 (3)	C12—C11—H11A	120.0
C2—C1—H1B	119.7	C11—C12—C13	119.3 (3)
C6—C1—H1B	119.7	C11—C12—H12A	120.3
C7—N2—N1	112.3 (2)	C13—C12—H12A	120.3
C7—N2—C10	130.2 (2)	C14—C13—C12	120.3 (3)
N1—N2—C10	117.04 (19)	C14—C13—H13A	119.8
C17—O2—C18	116.7 (3)	C12—C13—H13A	119.8
C1—C2—C3	120.4 (3)	C15—C14—C13	120.2 (3)
C1—C2—H2A	119.8	C15—C14—H14A	119.9
C3—C2—H2A	119.8	C13—C14—H14A	119.9
C4—C3—C2	118.9 (3)	C14—C15—C10	119.2 (2)
C4—C3—H3A	120.5	C14—C15—H15A	120.4
C2—C3—H3A	120.5	C10—C15—H15A	120.4
C3—C4—C5	121.0 (2)	O1—C16—C17	113.1 (2)
C3—C4—Cl	119.5 (2)	O1—C16—H16A	109.0
C5—C4—Cl	119.5 (2)	C17—C16—H16A	109.0
C4—C5—C6	120.1 (2)	O1—C16—H16B	109.0
C4—C5—H5A	119.9	C17—C16—H16B	109.0
C6—C5—H5A	119.9	H16A—C16—H16B	107.8
C1—C6—C5	118.9 (2)	O3—C17—O2	125.8 (3)
C1—C6—C7	122.4 (2)	O3—C17—C16	125.1 (3)
C5—C6—C7	118.7 (2)	O2—C17—C16	109.1 (3)
N2—C7—C8	106.6 (2)	O2—C18—C19	107.1 (3)
N2—C7—C6	124.7 (2)	O2—C18—H18A	110.3
C8—C7—C6	128.7 (2)	C19—C18—H18A	110.3
C7—C8—C9	104.6 (2)	O2—C18—H18B	110.3
C7—C8—H8A	127.7	C19—C18—H18B	110.3
C9—C8—H8A	127.7	H18A—C18—H18B	108.5
N1—C9—O1	122.1 (2)	C18—C19—H19A	109.5
N1—C9—C8	113.6 (2)	C18—C19—H19B	109.5
O1—C9—C8	124.3 (2)	H19A—C19—H19B	109.5
C11—C10—C15	120.9 (2)	C18—C19—H19C	109.5
C11—C10—N2	119.3 (2)	H19A—C19—H19C	109.5
C15—C10—N2	119.7 (2)	H19B—C19—H19C	109.5
			
C9—N1—N2—C7	−0.1 (3)	N2—N1—C9—C8	−0.9 (3)
C9—N1—N2—C10	−172.7 (2)	C16—O1—C9—N1	−8.7 (4)
C6—C1—C2—C3	0.2 (4)	C16—O1—C9—C8	170.3 (3)
C1—C2—C3—C4	−1.3 (4)	C7—C8—C9—N1	1.6 (3)
C2—C3—C4—C5	2.0 (4)	C7—C8—C9—O1	−177.5 (2)
C2—C3—C4—Cl	−176.5 (2)	C7—N2—C10—C11	134.0 (3)
C3—C4—C5—C6	−1.6 (4)	N1—N2—C10—C11	−55.0 (3)
Cl—C4—C5—C6	176.93 (19)	C7—N2—C10—C15	−49.0 (4)
C2—C1—C6—C5	0.2 (4)	N1—N2—C10—C15	122.0 (3)
C2—C1—C6—C7	−179.0 (2)	C15—C10—C11—C12	−1.1 (4)
C4—C5—C6—C1	0.4 (4)	N2—C10—C11—C12	175.8 (2)
C4—C5—C6—C7	179.7 (2)	C10—C11—C12—C13	2.0 (4)
N1—N2—C7—C8	1.0 (3)	C11—C12—C13—C14	−1.1 (5)
C10—N2—C7—C8	172.4 (2)	C12—C13—C14—C15	−0.6 (5)
N1—N2—C7—C6	−178.9 (2)	C13—C14—C15—C10	1.4 (4)
C10—N2—C7—C6	−7.6 (4)	C11—C10—C15—C14	−0.6 (5)
C1—C6—C7—N2	−45.5 (4)	N2—C10—C15—C14	−177.5 (2)
C5—C6—C7—N2	135.3 (3)	C9—O1—C16—C17	−70.7 (3)
C1—C6—C7—C8	134.5 (3)	C18—O2—C17—O3	−3.6 (4)
C5—C6—C7—C8	−44.7 (4)	C18—O2—C17—C16	174.0 (2)
N2—C7—C8—C9	−1.5 (3)	O1—C16—C17—O3	−16.5 (4)
C6—C7—C8—C9	178.5 (2)	O1—C16—C17—O2	165.9 (2)
N2—N1—C9—O1	178.1 (2)	C17—O2—C18—C19	−175.9 (3)
